# Fairness in the multi-proposer-multi-responder ultimatum game

**DOI:** 10.1371/journal.pone.0319178

**Published:** 2025-03-04

**Authors:** Hana Krakovská, Rudolf Hanel, Mark Broom

**Affiliations:** 1 Institute of the Science of Complex Systems, Center for Medical Data Science, Medical University of Vienna, Vienna, Austria; 2 Complexity Science Hub, Vienna, Austria; 3 Department of Mathematics, City, University of London, London, United Kingdom; Teesside University, UNITED KINGDOM OF GREAT BRITAINAND NORTHERN IRELAND

## Abstract

The Ultimatum Game is conventionally formulated in the context of two players. Nonetheless, real-life scenarios often entail community interactions among numerous individuals. To address this, we introduce an extended version of the Ultimatum Game, called the Multi-Proposer-Multi-Responder Ultimatum Game. In this model, multiple responders and proposers simultaneously interact in a one-shot game, introducing competition both within proposers and within responders. We derive subgame-perfect Nash equilibria for all scenarios and explore how these non-trivial values might provide insight into proposal and rejection behaviour experimentally observed in the context of one vs. one Ultimatum Game. Additionally, by considering the asymptotic numbers of players, we propose two potential estimates for a “fair” threshold: either 31.8% or 36.8% of the pie (share) for the responder.

## Introduction

The Ultimatum Game (UG) has been one of the paradigmatic games for studying fairness since its introduction in 1982 by Güth et al. [1]. This simple one-shot game consists of a reward and two players with asymmetric roles: proposer and responder. The proposer’s task is to suggest a split of the reward between the players. The responder can then accept or reject this split. On acceptance, the reward is split accordingly, and on rejection, both players receive nothing. The theoretical prediction states that a rational self-interested proposer will offer the minimum amount they believe the responder will accept. Similarly, a rational self-interested responder will accept any positive offer and remain indifferent between accepting or rejecting a zero offer. Thus, the subgame-perfect Nash equilibrium [2], dictates the proposer to offer the smallest possible unit of the reward which the responder then accepts.

However, in experiments, real-world players do not play as predicted by this theory (see e.g. Camerer [3] and Henrich et al. [4]). In Western, educated, industrialised, rich, democratic (W.E.I.R.D.) societies, responders reject offers of less than 20*%* of the reward with a probability of one-half and almost always accept proposals of 40*%* to 50*%* .  Proposers’ modal offers are usually between 40*%* to 50*%* and mean offers between 30*%* to 40*%* [3]. For example, Oosterbeek et al. [5] found in their comprehensive review a mean offer of 40*%* .  On the other hand, experimental results on UG-like scenarios (see again [4]), played in various small-scale societies, report more variable proposer and responder thresholds, ranging from greedy to generous. Interestingly, among the most generous proposers are tribal whaler societies [6], whose lifestyle requires high levels of cooperation and mutual trust. Elsewhere, it is common for low offers to be both proposed and accepted [7,8].

Additionally, the sensitivity to biases, including anonymity, stake level, and context effects have been studied, and it was concluded they do not seem to shift played thresholds significantly [9–11]. Moreover, *priming* players, e.g. by conjuring an imaginary right to play proposer, has been found to make players propose more greedily (see Hoffman et al. [12]), although some of the results have been recently contested by Demiral and Mollerstrom [13]. A concise understanding of how human sharing and cooperation behaviour precisely depends on context and circumstances (including the complexity of social interactions in terms of multi-player scenarios) is, therefore, still partly to be found.

Whether *fairness* is part of our ancestral behavioural makeup that evolved among apes or can be attributed only to the “modern” self-domesticated human [14,15] is still debated [16–19].

W.E.I.R.D. societies have been shown to follow a unique cognitive trajectory, concerning sharing and cooperation, which can be traced back to a change in inheritance law in 305 AD. It was introduced by the Catholic Church due to its preoccupation with incest, promoting heritage by testament, banning polygamous marriages and marriages to relatives and promoting the newlywed to set up independent households [20,21].

This reform, over a period of more than a thousand years, broke up clan structures and thereby fostered individuality over clan-identity, and the interaction and cooperation of individuals across clan-boundaries, which may explain some peculiarities of the W.E.I.R.D. world. In summary, our evolutionary and societal development remains partly veiled in the dusk of the past and can only be accessible through comparative studies (e.g. [14]) and to a *hypothetical realism* that largely remains grounded in mathematical modeling.

Evolutionary game theory is a common framework for studying the emergence of *fairness* (see e.g. Debove et al. [22] who review various evolutionary game-theoretic models of the UG type). For instance, some models on networks indicate a dependence of the proposer and responder thresholds on the topology of the social network (see e.g. Sinatra et al. [23], Kuperman and Risau-Gusman [24] and Page et al. [25]); others explore the role “social status”, may play [26]. Alternatively, Fehr and Schmidt [27] propose an inequity-aversion framework in their seminal work. Also, see the review by Güth and Kocher [28].

Regarding generalisations of the UG to multiple players, the majority of theoretical and experimental extensions typically focus on scenarios with either one proposer and many responders or vice versa. For instance, Roth et al. [29] conducted experiments with proposer competition and one responder, while Fischbacher et al. [30] explored responder competition. They showed that introducing competition raises the offers in the former and decreases offers in the latter as compared to the one vs. one UG.

Additionally, Santos et al. [31] investigated an evolutionary game-theoretic model with a group decision-making process for responders when faced with an offer from a single proposer. In another paper, Santos and Bloembergen [32] extend the UG to a group of proposers playing with a group of responders. The group of responders rejects the average proposed offer if it is lower than their average group acceptance threshold. In generosity or envy games there is an additional third dummy player that takes no active role (for details see survey from Güth and Kocher [28]).

In this paper, we introduce an extension of the UG where multiple proposers and multiple responders engage in a one-shot interaction. This extended model seems to be more appropriate in various social contexts, such as the tribal whaler society mentioned above than the classical two-player paradigm. It constitutes a two-sided market, where proposers simultaneously make their offers, and responders, in turn, simultaneously each select an offer from one of the proposers (or choose a number with some probability), or decline all offers. If a proposer is chosen by at least one responder, they receive the proposed split. Conversely, if no responders select them, they receive nothing. On the other hand, all responders who chose a proposer that was not selected by anyone else will receive their share. In cases where multiple responders choose the same proposer, only one of them, randomly selected, receives the proposed split, while the other responders receive nothing.

In the context of a simplified labour market, proposers represent potential employers and responders potential employees. We assume that the employers offer identical roles where the reward is the total revenue, and they must decide how to split it with the employee. If an employer chooses to claim too large a share of the revenue, they risk being outbid by other employers and earning nothing. Similarly, if the employees would simply choose the highest offer, they may end up making the same choice as many other responders, resulting in no job opportunity if they are not selected by the probabilistic rule. An essential factor influencing the “optimal” offers of employers is the balance between the number of available jobs (employers) and the number of potential employees. Alternatively, within a biological context, proposers may symbolise plants, offering their energy in the form of nectar, while responders represent the pollinators. The game is formulated as a one-shot interaction, meaning there is no sequential bargaining taking place. This is realistic under conditions where sequential bargaining is severely restricted e.g. by time or energy constraints.

A similar concept to the multiplayer UG is sequential bargaining (see e.g. Rubinstein and Wolinsky [33], Li et al. [34]). In these models, the proposer role is passed on around players until everyone agrees with the division. In Li et al. [34] they describe a model with two buyers and two sellers. Sequentially, each participant selects a partner from the opposing group and initiates bargaining by making an offer. If the initial pair reaches an agreement, they quit bargaining and leave with their share. However, in the case of rejection, they remain open to being chosen by another seller or buyer for further bargaining. If not all players agree on a share, in the subsequent round the other group takes the role of the proposer and makes the offers. However, these models differ due to their sequential quality and role switching, whereas in our model we present a one-shot model.

This paper is organised as follows. In the following section, we present a formalisation of the game and (for a self-contained description) briefly introduce the subgame-perfect Nash equilibria of the one vs. one and one vs. many UG scenarios. Then, in Two proposers and two responders we provide a detailed analysis of the game involving two proposers and two responders, where we find the subgame-perfect Nash equilibrium of the game. Subsequently, in K Proposers and L responders we derive the solution for the general case of multiple proposers facing multiple responders and perform an evolutionary simulation to compare the theoretical predictions with the simulations results. In the Discussion we scrutinise the discovered solutions, look closer at their asymptotic properties, and discuss some implications for fairness in human behaviour. Lastly, we summarise our results in the Conclusions.

## Model

In the following section, we extend the classical UG to a *Multi-Proposer-Multi-Responder* (MPMR) UG framework and, at the same time, comment on known results of three special cases: one responder vs. one proposer, many responders vs. one proposer, and one responder vs. many proposers.

The MPMR UG involves *L* ∈ *ℕ* responders and *K* ∈ *ℕ* proposers. Each proposer is endowed with a potential reward of size one that is to be split with one of the responders. The game has two stages. In the first stage, each proposer puts forward a split of the reward denoted as $s_{i}\in [0,1]$, where *i* ∈ { 1 , 2 , *…* , *K* } , offering $s_{i}$ to the responders and $1-s_{i}$ to themselves. All responders have full information about the offers. In the second stage of the game, responders simultaneously and independently (without the knowledge of other responders’ choices) select one of the proposers or select no one. Additionally, they may also use mixed strategies and probabilistically decide between multiple proposers. Responders have full information about all the proposed offers and are impartial towards the proposers themselves. Following the decision of the responders, the payoffs are distributed. If the proposer *i* was selected by at least one responder, they receive $1-s_{i}$, otherwise, they receive nothing. Similarly, only one responder picked randomly (with probability one divided by the number of individuals that chose the same proposer) among the respective responders receives the offered split $s_{i}$, the others receive nothing, just as responders who did not choose any proposer would.

Let us rewrite the payoffs in symbolic terms. Denote $N_{R,i}\in \{0,1,\ldots ,L\},$ *i* ∈ { 1 , 2 , *…* , *K* }  as the number of responders that chose the proposer *i*. The payoff $\pi_{P,i}$ of proposer *i* with offer $s_{i}\in [0,1]$ is calculated as:


$$\pi_{P,i}=\{\begin{array}{c} \begin{array}{ccccc} & 1-s_{i} & \;\text{if} & N_{R,i}\geq 1\;, & \\ & 0 & \;\text{if } & N_{R,i}=0\;. \end{array}\; \end{array}$$

The payoff $\pi_{R,j}$ of the responder *j* , *j* ∈ { 1 , 2 , *…* , *L* }  that chose proposer *i* is given as


$$\pi_{R,j}=\{\begin{array}{c} \begin{array}{ccccc} & s_{i} & \; & \text{with probability }\frac{1}{N_{R,i}}\;, & \\ & 0 & \; & \text{with probability }\frac{N_{R,i}-1}{N_{R,i}}\;. \end{array}\; \end{array}$$

The payoff $\pi_{R,j}$ of the responder *j* who rejected all offers is given as:


$$\pi_{R,j}=0\;.$$

**Fig 1:**
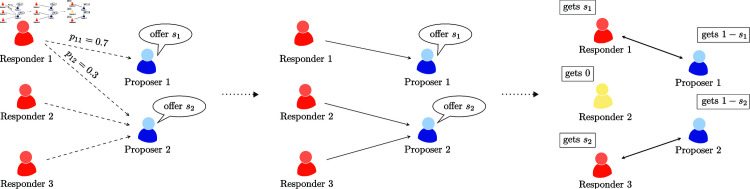
Graphical example of the MPMR UG for the case of *L* = 3 responders and *K* = 2 proposers. In the first stage, proposers announce their offers, prompting each responder to determine their selection strategy. In this scenario, responder 1 chooses a mixed strategy while the other two responders play pure strategies. In the second stage, it is probabilistically decided that responder 1 chooses proposer 1. Since two responders chose proposer 2 ,  another probabilistic realisation determines who gets paired with the proposer. In this case, responder 2 is unpaired, resulting in a zero payoff. Similarly, if one of the proposers (or both) would not be selected they would receive a zero payoff.

In Fig 1 we illustrate the mechanics of the MPMR UG in a simple scenario.

Before going into further analysis let us first summarise the known results for three basic cases of the game. With one proposer and one responder, the game reduces to a classical UG. If the responder’s strategy is to refuse any offer other than *s* , *s* ∈ [ 0 , 1 ]  then the proposer offering *s* corresponds to a Nash equilibrium. Likewise, it constitutes a Nash equilibrium if we presume that once a responder accepts *s*, they will accept any offer $s^{*}$ greater than *s* .  Consequently, there exists a continuum of Nash equilibria in the UG. To reduce their number, attention can be directed towards the concept of a subgame-perfect Nash equilibrium [2]. If the proposer deviates from their strategy and offers slightly less than *s*, in this subgame, the responder’s best-reply is to accept the offer. Consequently, no *s* > 0 can be a subgame-perfect equilibrium, since the proposer can always improve their payoff by lowering the offer. Thus, there exists a unique subgame-perfect equilibrium where the proposer offers *s* = 0 and the responder accepts all offers. If there exists a grid of possible offers instead of a continuous interval, the second smallest possible offer *s* = 0 is also a subgame-perfect equilibrium. This is because the responder maximises their payoff regardless of accepting or rejecting the offer of *s* = 0 and may choose to reject a zero offer, in which case the proposer’s subgame-perfect equilibrium is to offer *s* = 0

Similarly, in the case of multiple responders facing a single proposer, in the subgame-perfect equilibrium responders are offered zero and at least one of them accepts the offer. Once again, in the case of the existence of the second smallest offer, this is a subgame-perfect equilibrium too if all responders reject the zero offer. In the case of proposer competition, where multiple proposers face a single responder, in the subgame-perfect equilibrium at least two proposers offer *s* = 1 which the responder accepts (note that the other proposers can offer any split, which subsequently leads to multiple equilibria). For details see e.g. [27].

In the next sections, we will see that the introduction of two-sided competition yields intriguing outcomes. Since only one responder gets their share of the reward if more than one of them select the same proposer, it may not always be optimal for responders to straightforwardly select the best proposal, as others might employ the same approach. Instead, a more eﬃcient strategy might involve granting a non–zero probability of going to the second-best proposal and other alternative offers. In this sense, the two-sided competition problem introduces a minority-game-like situation (as in the El Farol Bar problem [35]) into the MPMR UG scenario. Consequently, the proposers are motivated to make offers below the offer of one, as there is a possibility that the second-best offer might still be accepted by some responder. On the other hand, there is still the presence of competition between the proposers which prevents them from offering zero.

In the next section, we start with the scenario involving two proposers and two responders.

## Two proposers and two responders

In this section, we provide an analysis of the MPMR UG with two proposers and two responders. Our goal is to find subgame-perfect Nash equilibria. We will see that in each subgame with at least one positive offer there are either one or two Nash equilibria for the responders, depending on the combination of offers. Only one of them is also an evolutionarily stable strategy (ESS) and when restricting ourselves to that strategy there is a unique subgame-perfect Nash equilibrium where both proposers offer *s* = 0 . 5 and both responders select each of them with probability 0 . 5 . 

### Responders’ Nash equilibrium

Let us start with denoting the strategy of one particular subgame (i.e. how much is offered to the responders) for the first proposer as $s_{1}\in [0,1]$ and for the second proposer as $s_{2}\in [0,1].$ Without loss of generality we assume $s_{1}\leq s_{2}.$ The mixed strategies of the respective responders (denoted $R_{1}$ and $R_{2}$) selecting the respective proposers (denoted $P_{1}$ and $P_{2}$) with their proposed offers $s_{1}$ and $s_{2},$ which are fixed in this subgame, will be given as:


$$\begin{array}{cccc} P(R_{1}\text{ choosing }P_{1}) & =p,\;P(R_{1}\text{ choosing }P_{2}) & =1-p\;,\\ P(R_{2}\text{ choosing }P_{1}) & =q,\;P(R_{2}\text{ choosing }P_{2}) & =1-q\;, & \end{array}$$

where *p* , *q* ∈ [ 0 , 1 ] .  Thus responder’s strategy depends on the proposed values– it is expressed as the probability of choosing the first proposer with a given offer $s_{1}$ or the second proposer with a proposed offer $s_{2}.$ Furthermore, in the analysis of finding the best reply strategy, we do not consider strategies where responders reject both positive offers, since these are redundant and will not constitute a best reply strategy (the only exception is if both proposers offer zero which is analysed below).

Considering these strategies we can calculate the expected payoffs of responders, denoted by $\Pi_{R,1},\Pi_{R,2}$ for the first and second responder respectively:


$$\begin{array}{ccc} \Pi_{R,1}(p,q) & =s_{1}p\left(1-q+\frac{q}{2}\right)+s_{2}(1-p)\left(q+\frac{1-q}{2}\right), & \\ \Pi_{R,2}(p,q) & =\Pi_{R,1}(q,p)\;. \end{array}$$

Next, we want to find the best response of the first responder given a set of offers $\left(s_{1},s_{2}\right)$ and a fixed mixed strategy  ( *q* , 1 − *q* )  of the second responder. We start by finding *p* that maximises $\Pi_{R,1}.$ Notice the payoff $\Pi_{R,1}$ is linear in *p* .  Thus, the derivative of $\Pi_{R,1}$ with respect to *p* is constant for all *p* and given as:


$$\begin{array}{c} \frac{\partial \Pi_{R,1}}{\partial p}=s_{1}-\frac{s_{2}+qs_{1}+qs_{2}}{2}. \end{array}$$ (1)

It is easy to find the best response strategy of the first responder, denoted by $p^{*}:$


$$\begin{array}{c} p^{*}=\{\begin{array}{c} \begin{array}{ccccc} & [0,1]\; & \text{if}s_{1} & =s_{2}=0\;, & \\ & [0,1]\; & \text{if}q & =q_{\text{crit}}\equiv \frac{2s_{1}-s_{2}}{s_{1}+s_{2}}\;,\\ & 1\; & \text{if}q & <q_{\text{crit}}\;,\\ & 0\; & \text{if}q & >q_{\text{crit}}\;. \end{array}\; \end{array} \end{array}$$ (2)

We would get mirror results for the best response strategy of the second responder since the responder roles are symmetric. It is evident from Eq (2), that when the higher offer $s_{2}$ exceeds twice the value of $s_{1}$, it is advantageous for both responders to disregard the lower offer $s_{1}.$ Conversely, in instances where the offers are suﬃciently similar ($s_{2}\leq 2s_{1}$), a critical threshold $q_{\text{crit}}$ emerges. If the second responder plays the threshold strategy $q_{\text{crit}}$, the first responder’s strategy becomes irrelevant. If the responder plays a different strategy to $q_{crit},$ the best response is to choose exclusively the other relatively less occupied proposer with respect to the threshold. As a consequence, the Nash equilibrium for responders in the subgame with fixed offers $s_{1}\leq s_{2}$ is:


$$\begin{array}{c} \begin{array}{cccccc} & \text{Scenario 0:}\; & \text{if} & s_{1}=s_{2}=0\; & p_{0},q_{0}\in [0,1]\text{ or reject .}\\ & \text{Scenario A:}\; & \text{if} & s_{2}<2s_{1}\; & p_{A}=q_{A}=\frac{2s_{1}-s_{2}}{s_{1}+s_{2}}\;. & \\ & \text{Scenario B:}\; & \text{if} & s_{2}<2s_{1}\; & p_{B}=0,\;q_{B}=1\text{ or reversed .} & \\ & \text{Scenario C:}\; & \text{if} & s_{2}=2s_{1}\neq 0\; & p_{C}=0,q_{C}\in [0,1]\text{ or reversed .} & \\ & \text{Scenario D:}\; & \text{if} & s_{2}>2s_{1}\; & p_{D}=q_{D}=0\;. & \end{array} \end{array}$$ (3)

In *Scenario 0*, both proposers offer zero and responders receive a zero payoff, no matter which strategy they choose. In *Scenario D* one of the offers is more than double the amount proposed by the other proposer. Thus, both responders opt for the proposer with the higher offer and discard the other. In the subgame with *Scenario C*, there is a continuum of Nash equilibria, however, playing the strategy of going to the second proposer with offer $s_{2}$ is a weakly dominant strategy (this is easy to see since the payoff is given as $\Pi_{R,1}=s_{1}(1\;+\;q\;-\;\frac{3}{2}pq)$). Notice that when the ratio of the offers is less than two ($s_{2}<2s_{1}$), two types of Nash equilibrium strategies for the responders emerge: symmetric strategies (*Scenario A*) and strategies which require “coordination” of the responders (*Scenario B*). Let us now look closer at how these strategies perform when played against each other. We denote the offer levels as $s_{1}=s,$ where *s* ∈ [ 0 , 1 ]  and $s_{2}=s\;+\;\delta ,$ where 0 ≤ *δ* < *s* and $0<s_{2}\leq 1.$ The payoff matrix for responders playing strategies $p_{A},p_{B}$ against $q_{A},q_{B}$ is shown in Table 1. Choosing the lower offer with probability zero ($p_{B}=0$) is the best response against both $q_{A}$ and $q_{B}=1,$ however when played against the same strategy $q_{B}=0$ the strategy is inferior to $p_{A}$ and $p_{B}=0.$ Thus, contrary to *Scenario C*, there is no weakly dominant strategy. In order to determine which of the two Nash equilibria will be played by the responders, we can turn to the concept of an evolutionarily stable strategy. As we prove in S1 Appendix (see Theorem 1) the symmetric strategy *p_A_* is uniquely evolutionarily stable, which means that it is (in this way) superior to other strategies.

**Table 1:** Payoff matrix for chosen strategies in *Scenario A* and *B*. Here we show the payoff matrix for responders playing strategy $p_{A},p_{B}= 1$ and $p_{B}= 0$ in *Scenario A* and *Scenario B.*

	$q_{A}$	$q_{B}= 0$	$q_{B}= 1$
$p_{A}$	$\frac{3s(s+\delta )}{2(2s+\delta )}$	$\frac{3s(s+\delta )+2\delta (\delta -s)}{2(2s+\delta )}$	$\frac{3s(s+\delta )+2\delta (2\delta +s)}{2(2s+\delta )}$
$p_{B}=0$	$\frac{3s(s+\delta )}{2(2s+\delta )}$	$\frac{1}{2}(s+\delta )$	*s* + *δ*
$p_{B}=1$	$\frac{3s(s+\delta )}{2(2s+\delta )}$	*s*	$\frac{1}{2}s$


We also explored the replicator dynamics with the co-existence of three types of Nash equilibrium strategies: *γ*–players who always choose the strategy of *Scenario A*, *α*–players who always choose the highest offer and *β*–players who always choose the lowest offer. The analysis, detailed in S1 Appendix, reveals that in the stable state *α*– and *β*–players’ relative abundances on average yield the same strategy as the *Scenario A* strategy, albeit on a population-wide level rather than an individual one. The abundance of *γ*–players depends on the initial conditions. Thus, in the next part, we reduce the analysis to responders playing the Nash equilibrium strategy $p_{A}.$

### Proposers’ Nash equilibrium strategy

In this section, we will derive the subgame-perfect Nash equilibrium of the proposers. Without loss of generality, we assume that $s_{1}\leq s_{2}.$ As demonstrated in the preceding section, in the cases where $2s_{1}\leq s_{2},$ both responders choose the proposer with the higher offer and the other proposer receives no reward. Since the abandoned proposer can improve their payoff by increasing their offer to slightly more than half of the other offer, this situation cannot be a Nash equilibrium. For the case where $s_{1}=s_{2}=0$, there is a subgame-perfect Nash equilibrium. Here both responders receive zero, no matter what their strategy is. If each responder accepts and chooses different proposers, then both proposers receive the maximal payoff of one. However, this requires responders to accept a zero reward and align, i.e. to match their choices and can be considered degenerate. Any other matching (or rejection) does not lead to a subgame-perfect Nash equilibrium. Then one of the proposers (or both) has an expected payoff of less than one and by raising their offer from zero to a suﬃciently small offer *γ*, they are able to secure a better payoff, *γ*.

In the following, we will only consider offers satisfying $0<s_{2}\leq 2s_{1}$. The expected payoffs of proposers 1 and 2 ,  with offers $s_{1}$ and $s_{2}$ respectively (denoted $\Pi_{P,1}$ and $\Pi_{P,2}$ respectively), are:


$$\begin{array}{ccc} \Pi_{P,1} & =(1-s_{1})\left(pq+p(1-q)+q(1-p)\right)\;, & \\ \Pi_{P,2} & =(1-s_{2})((1-p)(1-q)+p(1-q)+q(1-p))\;, \end{array}$$

where  ( *p* , 1 − *p* )  and  ( *q* , 1 − *q* )  are the responders’ strategies. We assume that the responders choose the strategy $p_{A}$ (see Eq (3)) according to the Nash equilibrium *Scenario A* (for reasons why we do not focus on *Scenario B* strategy see above). Then we can rewrite the expected payoffs as:


$$\begin{array}{c} \begin{array}{ccc} \Pi_{P,1} & =\frac{3s_{2}(2s_{1}-s_{2})(1-s_{1})}{{\left(s_{1}+s_{2}\right)}^{2}}\;, & \\ \Pi_{P,2} & =\frac{3s_{1}(2s_{2}-s_{1})(1-s_{2})}{{\left(s_{1}+s_{2}\right)}^{2}}\;. \end{array} \end{array}$$ (4)

To maximise the payoffs we set $\partial \Pi_{P,i}∕\partial s_{i}=0$, with *i* = 1 , 2, identify the best response of the proposers, yielding for the first and second proposer:


$$s_{1}^{*}=f(s_{2})=\frac{s_{2}^{2}+4s_{2}}{5s_{2}+2}\;\text{ and}\;s_{2}^{*}=f(s_{1})=\frac{s_{1}^{2}+4s_{1}}{5s_{1}+2}\;.$$

The subgame-perfect Nash equilibrium $\left(s_{1}^{*},s_{2}^{*}\right)$ has to satisfy $s_{1}^{*}=f(f(s_{1}^{*}))$ leading to a fourth order polynomial that has four roots $\{0,\frac{1}{2},-3+\sqrt{7},-3-\sqrt{7}\}.$ However, the only physical, non-trivial solution is $\frac{1}{2}$, which leads to the unique non-degenerate Nash equilibrium of symmetric offers $(\frac{1}{2},\frac{1}{2}).$ In that case, the payoffs of the players are the same in both roles:


$$\begin{array}{c} \begin{array}{c} \Pi_{R,1}=\Pi_{R,2}=\Pi_{P,1}=\Pi_{P,2}=\frac{3}{8}\;. \end{array} \end{array}$$ (5)

Finally, we remark on a potentially counter-intuitive observation: by changing the rules of the game and giving the responders a chance to coordinate for maximising their common payoff in each subgame, their subgame-perfect Nash equilibrium payoffs are lower than in the MPMR UG.

It is easy to see this from the following. Opting for the strategy where one responder selects the first proposer while the other selects the second, results in the responders receiving all of the available reward in every subgame, so more than with any other strategy. Then, the payoffs of the proposers are:


$$\begin{array}{c} \begin{array}{ccc} \Pi_{P,1}^{*} & =1-s_{1}\;,\;\Pi_{P,2}^{*} & =1-s_{2}\;. \end{array} \end{array}$$ (6)

However, then the subgame-perfect equilibrium is  ( 0 , 0 )  and the responders accept, since, under all offer combinations, the proposers receive their offered split and they have no incentive to raise the offers from zero. Thus, this type of coordination leads to worse outcomes for the responders.

## K Proposers and L responders

Now to the general case of *K* ∈ *ℕ* proposers and *L* ∈ *ℕ* responders where *K* , *L* ≥ 2 . 

### Responders’ strategy

Let us find the Nash equilibria for the responders for subgames determined by offers from *K* proposers. Let $p_{ij}=P(R_{i}\;\text{choosing}\;P_{j})$, *i* ∈ { 1 , 2 , *…* , *L* } , with *j* ∈ { 1 , 2 , *…* , *K* } , denote the probability of the responder *i* choosing proposer *j*, who offers $s_{j}\in [0,1].$ Naturally, $\sum_{j=1}^{K}p_{ij}=1$ and $p_{ij}\geq 0$ and the expected payoff $\Pi_{R,l}$ of the responder *l* is:


$$\Pi_{R,l}=\overset{K}{\underset{i=1}{\sum }}s_{i}W_{li}\cdot p_{li}\;,$$

where


$$W_{li}=\overset{L}{\underset{j=1}{\sum }}\frac{1}{j}\left[\underset{\alpha :{\left\|\alpha \right\|}_{1}=j,\alpha_{l}=1}{\sum }\overset{L}{\underset{k=1,k\neq l}{\prod }}p_{ki}^{\alpha_{k}}{\left(1-p_{ki}\right)}^{1-\alpha_{k}}\right]\;,$$

with $\alpha \in {\left\{0,1\right\}}^{L}$ and ${\left\|.\right\|}_{1}$ being the $L^{1}$ norm. Each term of the sum in *W_li_* describes the probability of precisely *j* responders (including responder *l*) choosing proposer *i* ,  weighted by $\frac{1}{j}.$ Also notice that $W_{li}$ is independent of $p_{li},$ meaning that payoff $\Pi_{R,l}$ is a linear function defined by strategy $p_{l}=(p_{l1},p_{l2},\ldots ,p_{lK})$ and scalars $(s_{1}W_{l1},s_{2}W_{l2},\ldots ,s_{K}W_{lK}).$ In order to find the best response of responder *l* facing the other responders, we have to solve a constrained optimisation problem on a linear function $\Pi_{R,l}.$ Without loss of generality, we set the ordering of proposers to be determined by:


$$s_{K}W_{lK}\geq s_{i}W_{li}\;,$$

for all *i* ∈ { 1 , 2 , *…* , *K* }  and analyse the derivative of the payoff of responder *l* with respect to $p_{li},i\in \{1,\ldots ,K-1\}$ where $p_{lK}=1-\sum_{j=1}^{K-1}p_{lj}:$


$$\begin{array}{c} \begin{array}{c} \frac{\partial \Pi_{R,l}}{\partial p_{li}}=s_{i}W_{li}-s_{K}W_{lK}\;. \end{array} \end{array}$$ (7)

Thanks to the ordering, we know the derivative (see Eq (7)) has only non-positive values. The best response strategy is to set as zero all $p_{li}$ for which $\frac{\partial \Pi_{R,l}}{\partial p_{li}}<0$ and when determining the remaining probabilities where $\frac{\partial \Pi_{R,l}}{\partial p_{li}}=0$ the responder is ambivalent. Translating these best responses to Nash equilibria for each subgame is more intricate in the higher-dimensional scenario than in the simpler two vs. two case.

Once again, there is a *Scenario 0*-like regime where all proposers offer zero and the responders’ strategy is inconsequential– responders receive zero in all cases. There are also regimes similar to *Scenarios C* and *D* where some of the proposers offer too little compared to the other proposers and their offers get discarded by every responder. In regimes where all offers are suﬃciently similar to each other, both symmetric and asymmetric Nash equilibria resembling *Scenario A* and *Scenario B* can emerge, where each proposer has a positive probability of being selected. As an example, consider the case of two responders and three proposers offering identical offers *s* ∈ ( 0 , 1 ] .  Let $p=(p_{11},p_{12},p_{13})$ and $q=(p_{21},p_{22},p_{23})$ represent the responders’ probabilities for selecting the proposers. Both *p* = *q* = ( 1 ∕ 3 , 1 ∕ 3 , 1 ∕ 3 )  and *p* = ( 1 , 0 , 0 )  and *q* = ( 0 , *x* , 1 − *x* )  with *x* ∈ [ 0 , 1 ]  constitute Nash equilibria. Thus, a continuum of Nash equilibria exists, contrary to the previous section, where only two Nash equilibria emerge in the case of identical offers.

Due to these intricacies, we refrain from explicitly deriving parameter regions and all possible Nash equilibria for the responders. Instead, we restrict our analysis to Nash equilibria for the responders that are evolutionarily stable. For that, we consider an infinite population of responders from which we choose the respective number of players in each subgame. Thus, the players cannot coordinate. In Theorem 2 (see S1 Appendix, “General Case”), we prove that for any combination of offers $\left(s_{1},s_{2},\ldots ,s_{K}\right)$, apart from purely zero offers ($s_{i}=0,$ for all *i* ∈ { 1 , 2 , *…* , *K* } ), there exists a unique evolutionarily stable strategy for the responders. We will consider this responder strategy to be the “superior” Nash equilibrium, in the sense of *evolutionary stability*. Now we will show some implicit results about the evolutionarily stable Nash equilibrium.

Without loss of generality, let $s_{K}\geq s_{K-1}\geq \cdots \geq s_{1}$ with $s_{K}>0$ represent the offers from *K* ≥ 2 proposers. Assume that all responders employ the evolutionarily stable strategy ${\left\{p_{i}\right\}}_{i=1}^{K}$. When inserting the same strategy $p_{li}=p_{i}$ for all *l* ∈ { 1 , 2 , … , *L* } ,  *i* ∈ { 1 , 2 , *…* , *K* }  in the derivative (see Eq (7)), rewriting it and utilising the binomial formula, we obtain the same derivative of the payoff for all responders (denoted $\Pi_{R}$):


$$\begin{array}{c} \begin{array}{cc} \frac{\partial \Pi_{R}}{\partial p_{i}} & =\frac{1}{L}(s_{i}f(p_{i})-s_{K}f(p_{K}))\;, \end{array} \end{array}$$ (8)

where *i* ∈ { 1 , 2 , *…* , *K* − 1 }  and *f *(*x*) is defined as:


f:[0,1]→ℝ,f(0)=L,f(x)=L∑j=1L1jL−1j−1xj−1(1−x)L−j=1−(1−x)Lx.

The function *f * is continuous and strictly decreasing on  [ 0 , 1 ]  (see Lemma 1 in S1 Appendix). Since $s_{K}$ is the highest offer, $p_{K}$ has to be greater than zero in the Nash equilibrium, which means no other $p_{i}$ can be equal to one. Therefore, all derivatives (8) must be zero or negative in the Nash equilibrium. From this we know, that the evolutionarily stable strategy ${\left\{p_{i}\right\}}_{i=1}^{K}$ has to follow for all *i* ∈ { 1 , 2 , *…* , *K* − 1 }  that:


$$\begin{array}{c} \begin{array}{ccccc} & p_{i}=0 & \text{and}\; & s_{i}f(p_{i})-s_{K}f(p_{K})\leq 0\;, & \\ \text{or} & \\ & p_{i}>0 & \text{and}\; & p_{i}=f^{-1}\left(\frac{s_{K}}{s_{i}}f(p_{K})\right)\;. \end{array} \end{array}$$ (9)

where in the latter case $p_{i}$ is given as a solution to $\frac{\partial \Pi_{R}}{\partial p_{i}}=0.$ Note, it is clear that when $s_{i}=0,$ $p_{i}$ has to be zero too.

Next, we show that for any offer combination (apart from pure zero offers) there exists a unique solution that follows Eq (9). For this, we will construct a function *h* that measures the sum of solutions that follow Eq (9) for every fixed $p_{K}$ and show that there is only one possible solution that sums to one.

Since the function *f * is strictly decreasing on  [ 0 , 1 ]  it is also invertible on  [ 0 , 1 ] .  By fixing $p_{K}$ we can uniquely determine all $p_{i}$’s that follow Eq (9). They are given as $p_{i}=f_{0}^{-1}\left(\frac{s_{K}}{s_{i}}f(p_{K})\right),$ where $f_{0}^{-1}(x)=f^{-1}(x)$ on  [ 1 , *L* ]  and zero on  [ *L* , + *∞* ] .  Naturally, such $p_{i}$’s do not necessarily sum to one, but they must in the solution. In order to track this, we define a function *h* that describes the sum of the $p_{i}$’s that follow Eq (9) for a fixed $p_{K}$:


$$\begin{array}{c} h:[0,1]\to \mathbb{R},\;h(p_{K})=p_{K}+\overset{K-1}{\underset{i=1}{\sum }}f_{0}^{-1}\left(\frac{s_{K}}{s_{i}}f(p_{K})\right), \end{array}$$ (10)

To find the equilibrium strategy we need to find $p_{K}$ such that the sum $h(p_{K})=1.$ The function *h* is strictly increasing in *p_K_*. For $p_{K}=0:h(0)=0,$ for $p_{K}=1$ we have *h* ( 1 ) = 1 if $s_{K}\geq Ls_{K-1}$ and *h* ( 1 ) > 1 otherwise. Since *h* is continuous and monotone there has to be a unique value that satisfies $h(p_{K}^{*})=1.$ This solution is a Nash equilibrium and as we will show in the Theorem 2 (see S1 Appendix, “General Case”) it is also an evolutionarily stable strategy.

For a large number of proposers and responders, it is not possible to find a closed-form solution of the responders’ evolutionarily stable strategy (numerically it is possible e.g. by utilising the *h* function in Eq (10)), however, this does not prevent us from deriving the Nash equilibria of the proposers in the next section.

### Proposers’ strategy

In this section, we analyse the proposers’ subgame-perfect Nash equilibria. We assume that for each offer regime, all responders play with a unique evolutionarily stable strategy, found as a solution to Eq (9).

First, let us comment on the situation where all offers are zero; in this situation, the responders receive zero no matter what strategy they choose. If the number of proposers is bigger than the number of responders there exists no subgame-perfect Nash equilibrium. However, if *K* ≤ *L* we have degenerate equilibria where each of the *K* responders has to choose one of the *L* proposers with probability one and the other *K*–*L* responders may choose an arbitrary strategy. Then, all proposers earn a payoff one and cannot improve further. When the responders opt for strategies that lead to at least one proposer having a probability of selection less than one, this cannot be a subgame-perfect Nash equilibrium. The proposer who earns less than one could offer a suﬃciently small amount *K* ≤ *L*, leading to all responders selecting them and resulting in an improved payoff of *K* ≤ *L*.

Next, we focus on offer regimes where the highest offer is bigger than zero, i.e. where $s_{K}>0$. Offer regimes where some of the proposers end up with zero selection probabilities under responders’ evolutionarily stable strategy cannot constitute a subgame-perfect Nash equilibrium. The rationale behind this is straightforward: a proposer with a zero probability of being selected will earn no reward, and by simply offering a suﬃciently higher amount, any proposer can secure a positive payoff. If we had for any *i* :  $\frac{\partial \Pi_{R}}{\partial p_{i}}<0$ (see Eq (8) and Eq (9)), then this would give a zero selection probability for proposer *i*. Thus, we can restrict our search only to those regimes where the system of equations $\frac{\partial \Pi_{R}}{\partial p_{i}}$ from Eq (8) is equal to zero for all *i* ∈ { 1 , 2 , *…* , *K* − 1 }  and $p_{i}\neq 0$ for all *i* ∈ { 1 , 2 , *…* , *K* } .  Then it is true for all *i* ∈ { 1 , 2 , *…* , *K* − 1 }  that:


$$C:=\frac{s_{i}}{p_{i}}\left(1-{\left(1-p_{i}\right)}^{L}\right)=\frac{s_{K}}{p_{K}}\left(1-{\left(1-p_{K}\right)}^{L}\right).$$

Additionally, we know that the payoff of proposer *i* with offer $s_{i}$ is given as:


$$\begin{array}{c} \Pi_{P,i}=(1-s_{i})\left(1-{\left(1-p_{i}\right)}^{L}\right)\;. \end{array}$$

In order to find the subgame-perfect Nash equilibrium of proposers (for details see Theorem 3 in S1 Appendix, “General Case”) we look at the derivative:


$$\begin{array}{c} \begin{array}{cc} \frac{\partial \Pi_{P,i}}{\partial s_{i}} & =\left(\frac{g_{i}^{2}C}{s_{i}^{3}H}-\frac{g_{i}C}{s_{i}^{2}}\right)\left(L{\left(1-p_{i}\right)}^{L-1}-C\right)-\frac{p_{i}g_{i}C}{s_{i}^{2}H}\;, \end{array} \end{array}$$ (11)

where $g_{i}=\frac{p_{i}^{2}}{{\left(1-p_{i}\right)}^{L-1}(Lp_{i}+1-p_{i})-1}$ and $H=\sum_{j=1}^{K}\frac{g_{j}}{s_{j}}$. In the equilibrium, the derivative in Eq (11) has to be equal to zero for all *i* ∈ { 1 , 2 , *…* , *K* } .  In Theorem 3 (see S1 Appendix) we show that such solutions must be symmetric, i.e. $s_{i}=s,s\in (0,1)$ for all *i* ∈ { 1 , 2 , *…* , *K* } .  In that case also the selection probabilities of responders are symmetric $p_{i}=p=\frac{1}{K}$ for all *i* ∈ { 1 , 2 , *…* , *K* } ,  since this solution satisfies the conditions for the unique responders’ ESS strategy. Taking all this into account, solving the system in Eq (11) being equal to zero is equivalent to solving:


$$\begin{array}{ccc} 0 & =\left(\frac{g}{s}-H\right)\left(L{\left(1-p\right)}^{L-1}-C\right)-p\;, & \end{array}$$

where $g=\frac{p^{2}}{{\left(1-p\right)}^{L-1}(Lp+1-p)-1}$, $H=K\frac{g}{s}$ and $C=\frac{s}{p}\left(1-{\left(1-p\right)}^{L}\right)$. From this we can derive the unique solution $s^{*}:$


$$\begin{array}{cc} s^{*} & =\frac{g(1-K)L{\left(1-p\right)}^{L-1}}{g(1-K)\frac{1-{\left(1-p\right)}^{L}}{p}+p}\;. \end{array}$$

By submitting $p=\frac{1}{K}$ we get:


$$\begin{array}{c} \begin{array}{cc} s^{*} & =\frac{L{\left(K-1\right)}^{L}}{K^{L+1}-{\left(K-1\right)}^{L-1}((K-1)K+L)}. \end{array} \end{array}$$ (12)

It is easy to show that $s^{*}\in (0,1)$ for all *K* , *L* ≥ 2 and that the second derivative of Eq (11) is always negative (see Proposition 1 in S1 Appendix). Therefore, $s^{*}$ in Eq (12) is indeed a subgame-perfect Nash equilibrium under the assumption that responders behave according to the evolutionarily stable strategy. Apart from the degenerate case of all offers being equal to zero, this is the only subgame-perfect Nash equilibrium. The expected payoff of proposers and responders is:


$$\begin{array}{c} \begin{array}{ccc} \Pi_{P} & =(1-s^{*})\left(1-{\left(1-\frac{1}{K}\right)}^{L}\right), & \\ \Pi_{R} & =s^{*}\frac{K}{L}\left(1-{\left(1-\frac{1}{K}\right)}^{L}\right). \end{array} \end{array}$$ (13)

**Fig 2:**
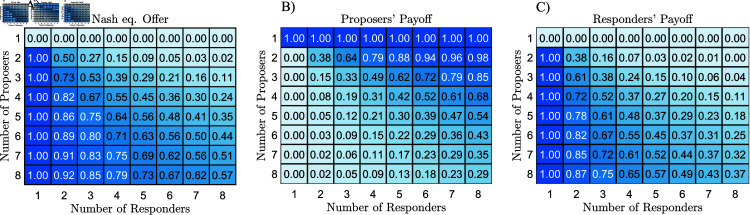
Numeric values of the proposers’ Nash equilibria and expected payoffs. (A) Numeric values of offers from Eq (12). (B) Expected payoffs of the proposers. (C) Expected payoff of the responders. See Eq (13) for the formulas. The values are shown for varying numbers of responders and proposers.

Next, we analyse the result in Eq (12) and investigate how the competition (im)balance of both sides of the market influences the thresholds $s^{*}$. Numeric values of equilibrium offers and equilibrium payoffs for a small number of proposers and responders are shown in Fig 2. One can notice, that for a fixed number of proposers ( ≥ 2) and an increasing number of responders, i.e. increasing responder competition, the equilibrium offers decrease. On the other hand, for a fixed number of responders ( ≥ 2) and increasing proposer competition, the proposers’ offers increase. This observation aligns with intuitive expectations about the effect of competition. The expected payoffs for responders and proposers follow the same trend. The proposers achieve the highest expected payoff when a single proposer faces any number of responders. Conversely, a responder gains the most when they are the sole responder facing at least two proposers.

Subsequently, we shall examine the Nash equilibrium strategy and payoffs of both proposers and responders in the asymptotic scenario where *K* ≫ 1 and *L* ≫ 1 and the ratio of the number of proposers to the number of responders is determined by a constant factor *c* > 0 ,  i.e. *K* = *cL* .  A low value of *c* means there is strong responder competition, high *c* implies strong proposer competition. Then the equilibrium $s^{*}$ depends on *c* as:


$$\begin{array}{c} \begin{array}{c} s_{c}^{*}=\frac{1}{c(e^{\frac{1}{c}}-1)}\;. \end{array} \end{array}$$ (14)

The payoff of the proposers and responders in this asymptotic situation is:


 lim ⁡ L→∞ΠP=1−c+1e1cc,lim ⁡ L→∞ΠR=1e1c. (15)

**Fig 3:**
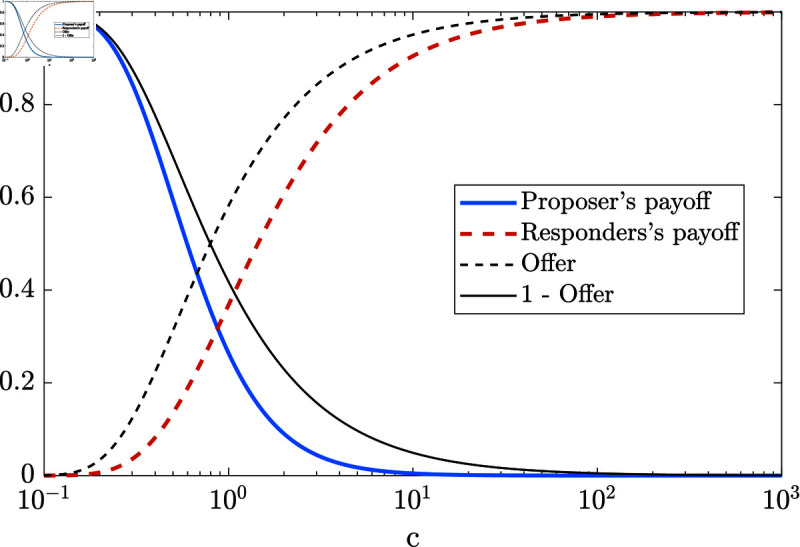
The expected payoffs and offer levels for varying proposer-responder ratio *c* . Here we show the expected payoff for proposers (blue), for responders (red dashed), the offer level (black) and 1-the offer level (black dashed) in subgame-perfect Nash equilibrium with respect to the proposer-responder ratio *c*, i.e. *K* = *cL*, in the large *L* limit (compare Eq (14) and Eq (15)). The differences between the offer and expected payoffs arise from “ineﬃciencies”. That is, with some probability some responders choose the same proposer and some proposers may not get chosen by any responder.

In Fig 3 we plot the results from Eq (14) and Eq (15). One can see how variations in the competition factor *c* impact the offers and payoffs in the limit.

In the next subsection, we will asses the agreement of the theoretical predictions with simulations of the evolutionary process. Subsequently, in the following section, we will look deeper into our analysis and explore possible implications of the MPMR UG on fairness.

### Evolutionary simulations

Thus far, we have focused on theoretical derivations, demonstrating the existence of unique evolutionarily stable solutions for responders and their corresponding subgame-perfect Nash equilibria of the MPMR UG. In this subsection, we will assess whether these solutions align with the predictions derived from basic evolutionary dynamics in a well-mixed population.

As before, we denote the number of proposers in the MPMR UG as *K* and responders as *L* .  The simulations start by initialising a population of 420 proposers with uniformly distributed offers on the interval  [ 0 , 1 ] .  Note that we selected 420 players as it is divisible by both 3 and 4 and therefore convenient for forming triples and quadruples in each round.

In each iteration, referred to as a *round*, every proposer plays one game with *K*–1 randomly selected proposers. For each game and the corresponding set of *K* offers, an evolutionary process for the responders is performed as described below. When this process is finished, *L* responders are randomly chosen from the evolved population, and through their selection the payoffs of the proposers are calculated, this selection is repeated until all responders played once. After each round, 10*%* of the proposers with the lowest payoffs are replaced via a noisy replication mechanism– they adopt the strategies of the 10*%* of the proposers with the highest payoff, with noise drawn from a uniform distribution on the interval  [ − 0 . 005 , 0 . 005 ] .  This evolutionary cycle is repeated for 1000 rounds.

A similar evolutionary process governs the responders’ strategies for a given set of *K* offers. The number of responders is 420 and their initial selection probabilities are drawn from a uniform distribution ${\left[0,1\right]}^{K}$ and then normalised to sum to one. To simplify the simulations, we exclude strategies with non-zero rejection probability as under the chosen simulation setup, they would inevitably die out in the early rounds. Each responder participates in 100 games per round and accumulates payoffs, interacting with randomly selected players. Following each round, 10*%* of the responders with the lowest payoff are replaced by noisy replicates of the 10*%* of the responders with the highest payoff. The noise of the replication is drawn again from a uniform distribution on the interval  [ − 0 . 005 , 0 . 005 ] .  This process is repeated for 100 rounds to allow for the evolution of responder strategies.

**Fig 4:**
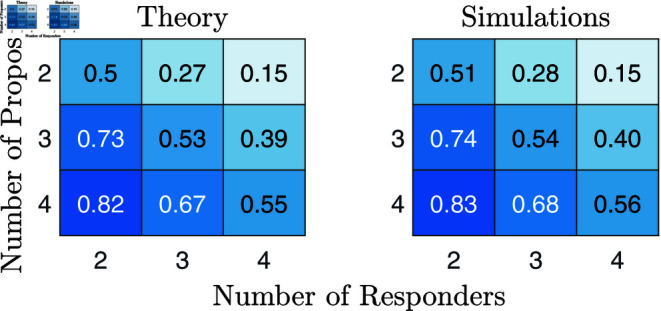
Comparison of theoretical predictions of proposers’ offers in the subgame-perfect Nash equilibrium and evolutionary simulation results. On the left, we show the theoretical predictions derived in the previous sections, on the right the results of the evolutionary simulations for 9 different scenarios with varying numbers of proposers and responders.

We conducted 5 independent simulation runs for each configuration, with combinations of 2 to 4 proposers and responders. The results and the theoretical predictions for given scenarios are summarised in Fig 4. They show a strong convergence to the theoretical predictions across all scenarios. Moreover, the standard deviation of the strategies of proposers is very low in the evolved population in all scenarios (lower than 0 . 015) and also of the means in-between the different runs (in the order of $10^{-3}$). Responders’ strategies have high standard deviation, but their average matches the evolutionarily stable strategy well.

Finally, we emphasise that in this formulation, the evolution of responders and proposers is decoupled, and there is no minimally acceptable offer– a slight departure from the standard setup in one vs. one Ultimatum Game simulations. This choice was made to simplify the strategy space of responders, which is more complex in the multi-proposer scenario and warrants further exploration in future research, especially with the introduction of a social network of the players. The consistency of the theoretical and simulated results indicates that the simplifying assumptions underlying the theoretical derivations do not impair their validity in the heterogeneous evolutionary setting.

In the following section, we will analyse and explore possible implications of the MPMR UG on fairness.

## Discussion

Throughout our evolutionary history, the interactions within social communities have played a crucial role in shaping the behaviour of our species. Many of these interactions were not interactions in pairs but rather in groups, suggesting that the MPMR UG with multiple players on both sides can contribute to the ongoing debate on *fairness* extending beyond the insights provided by the one vs. one UG. In this section, we will discuss some of these potential implications.

The perception of what is a *fair* or *equitable* division may depend on the properties of the community one is part of. Individuals presented with a particular opportunity may represent the proposers. Others may still be essential for task completion and act as responders. If responders perceive a shortage of proposers they may find it *fair* to accept a smaller share of the reward in exchange for assisting the proposer.

Then a central question arises: what is the underlying sense of group size balance between proposers and responders (i.e. proposer-responder ratio *c*) in the (local) community? And consequently, what would responders in our model deem a fair share of the reward in such a situation? A basic initial assumption could be a balanced scenario where the number of proposers equals that of responders, i.e. when *c* = 1 .  In this case, the subgame-perfect Nash equilibrium offer $s_{1}^{*}$ is equal to  ≈ 0 . 582 ,  which is slightly above the equality threshold of 0 . 5 .  The expected payoff of responders is  ≈ 0 . 368 and of proposers is  ≈ 0 . 264 (see Eq (14) and Eq (15)). When a responder is directly approached by a proposer (resembling the one vs. one UG), they may deem it fair to receive a share of the reward equivalent to what they would obtain in the community setting of the MPMR UG, i.e. 36.79% of the reward.

However, when *c* = 1, a proposer receives a smaller payoff than a responder. In this situation being a proposer is less beneficial. From an evolutionary perspective, we could anticipate that individuals would pursue the roles with larger payoffs until the payoff difference vanishes. Such an “equity” ratio *c* when the payoffs for members of both groups are identical is *c* = 0 . 872 ,  meaning there are fewer proposers than responders. The payoff of each proposer and responder is approximately 0 . 318 and the proposers’ offers are around 0 . 534 .  Once again, a responder could deem it fair to get the same payoff of 0 . 318 from the one vs. one interaction with a proposer as they would enjoy in the MPMR UG scenario with many players.

Note if there is some cost the proposer has to pay in order to be able to split and share the reward (e.g. energy and time invested to find the opportunity) their actual payoff is lower accordingly. This moves the blue curve of the proposers’ payoff in Fig 3 down, changing the equity ratio to a lower number, leading to an even smaller proportion of proposers in the community and finally smaller rewards in the equity state.

Next, let us compare the derived thresholds with the literature on the UG and the Dictator Game in which the responder cannot refuse the split and gets what is offered by the proposer. As an indication of fairness norms, one might look at the outcomes of the Dictator Game. Even though their offers cannot be refused, proposers in experiments often give a positive reward to the responder. In a meta-analysis by Engel [36] the mean offer in the Dictator Game was 28*%* with a modal (i.e. typical) offer of zero among W.E.I.R.D. societies. Conversely, in three small-scale societies, Henrich et al. [4] found mean offers of 20%, 31%, and 32% percent and only a few subjects offered zero. Another recent meta-analysis by Cochard et al. [37] supports the results with a calculated mean offer of 30.6% in the Dictator Game. We can notice these values are very close to our estimated payoff of the responder in the balanced case (31.8%).

In a meta-analysis of UG studies, Oosterbeek et al. [5] found a mean offer of 40*%* and Cochard et al. [37] found the mean to be 42 . 58*%* .  Henrich et al. [4] conducted experiments within small-scale societies and found the mean offers ranging from 26 % to 58% for different societies. One can ask whether proposers’ behaviour can be attributed to fairness concerns or simply strategic behaviour, wherein they offer proposals that maximise their payoffs given the assumed distribution of acceptance among responders. For example, Roth et al. [29] found evidence supporting strategic behaviour among proposers. On the other hand, Henrich et al. [4] concluded that offers tend to be higher than the optimal payoff-maximising offer, presumably due to pessimism regarding rejection frequencies and ambiguity aversion. The payoff maximising offers are found to be around 25%-40% for different small-scale societies. Nevertheless, Henrich et al. also noted instances where certain groups tended to accept nearly all low offers, while other groups commonly rejected high offers. Furthermore, Solnick [38] found the average minimally acceptable offer (the offer below which the responder rejects the offer) to be 30.8%. Once again, the thresholds are quite similar to the ones proposed from the MPMR UG.

Other authors, e.g. Schuster [39], claim the Golden Ratio, of about 0.618 vs. about 0.382, to be the solution for fair thresholds and support this claim by providing numerous examples from the literature that illustrate real-life situations exhibiting similar patterns. This threshold is once again close to ours, even though the author describes different mechanisms for achieving it.

Naturally, there are other possible extensions of the UG that we did not explore. For example, proposers could accept all responders who choose them, removing competition among responders and leading to proposers offering the full reward in the equilibrium. Another variation might involve splitting the reward evenly if multiple responders choose the same proposer, resulting in identical expected payoffs and the same equilibrium outcomes as in our formulation, or modeling cooperation in group tasks rather than pairs. However, we focused on the current setup as we believe it effectively captures the dynamics of competition and cooperation over scarce resources (or opportunities).

In summary, we believe the introduced extension of UG may hint toward mechanisms influencing the fairness norms in society. This applies both to personal relationships and broader public contexts, such as the labour or business market. In such settings, analyzing the balance– or imbalance– between proposers (employers) and responders (employees) could shed light on how competition for scarce employees or jobs affects wage fairness and employment terms. For example, in labour market situations where certain skills are scarce, our model predicts that the profit distribution between employer and employee will shift to favor the employee. Conversely, when skills are abundant, the distribution is likely to favour the employer.

## Conclusions

We have proposed a multi-player version of the UG, which we named the Multi-Proposer-Multi-Responder Ultimatum Game, with multiple responders and proposers playing simultaneously in a one-shot manner. Our work offers a new perspective on the UG with the interplay between proposer and responder competition. We analysed the responders’ strategy patterns and found that there can be a continuum of Nash equilibria of responders for some subgames (determined by offers from proposers), however, there is only one unique evolutionarily stable strategy in each subgame. We analytically derive subgame-perfect Nash equilibria of the proposers with respect to this strategy and find that situations with multiple proposers and responders in both groups lead to non-trivial offer values. These are unique for each parameter regime (except for the *degenerate* solutions of all offers being zero). We stress that the MPMR UG does not include bargaining sequential dynamics to reach non-trivial offer levels and we achieve this under a one-shot setting.

We test the theoretical predictions through simulations of an evolutionary model and find a strong agreement. While the simulations employ a simplified strategy space and replicator mechanism, they serve as both a valuable “sanity check” and a solid foundation for future research. Future studies could examine the effects of alternative replicator mechanisms, more refined strategy spaces, or the incorporation of interaction structures. In particular for the UG played on social networks, MPMR UG-like situations happen naturally in the neighbourhood of individuals. Conversely, the MPMR UG can explain the incentives for the way social networks evolve and restructure.

Admittedly, the game’s assumptions are heavily simplified when compared to complex economic and societal realities. We consider all offers to be of identical quality, all with the possibility to be reached in the same way, we assume all responders have full information of all the proposals and there is no sequential or repeated dynamics.

If decision-making is time-constrained and offering is sporadic, the latter two restrictions could become particularly relevant. In other scenarios, one might explore sequential or repeated bargaining. Repeated games, in particular, enable building reputation or coordination and punishment/reward dynamics, which can produce significantly different outcomes as compared to one-shot interactions (see e.g. the repeated donation game [40,41]). For instance, if responders were aware of which proposers other responders chose in previous rounds, this could facilitate coordination, prompting proposers to adjust their offers strategically. Similarly, proposers’ strategies would be affected by such setup. Introducing sequential elements, whether in the proposers’ offering order or responders’ selections, would add another layer of dynamic interactions. Another key factor is whether the roles are fixed or rotated. For example, in the latter scenario, Li et al. [34] suggest that with an equal aggregated discount factor on the reward, the equilibrium in a multiplayer bargaining game tends toward an equal split.

In another extension of this model, one could investigate the inclusion of additional heterogeneity among proposers or responders. For example, in levels of endowments among proposers or certain preferences among responders for specific proposers (e.g. due to geographical closeness, personal closeness etc.). This could shed light on the emergence of fairness norms in environments with inherent inequalities.

Yet, we believe there is an untapped potential in examining notions of fairness within the context of larger groups of players. Since real-world interactions rarely involve just two individuals, and the presence of external alternatives remains pertinent, we believe this expanded viewpoint can shed more light on our understanding of why fairness is perceived as it is.

Our analysis has revealed the potential impact of the ratio between the (asymptotic) number of proposers and responders on the perception of *fairness* in multi-player scenarios. We postulate that the sense of fairness within these scenarios then subconsciously influences people’s behaviour in the UG laboratory experiments. We compared these insights with the experimental literature of one vs. one UG and Dictator Game and found them to be of intriguing similarity. Despite its explanatory value, the presented MPMR UG model depends on many assumptions, and the reality of economic interactions is much richer which can probably to some extent bias the presented results.

Nonetheless, our mechanisms reproduce a relevant range of offer thresholds observed in experiments and we believe they are a noteworthy contribution to the ongoing discussion of fairness.

## Supporting information

S1 Appendix Detailed analysis and proofs of main theorems. We expand on the mathematical derivations and technical steps that were briefly outlined in the core sections of the paper. We provide detailed proofs and theorems that support the results presented in the main text.
(PDF)
