# Supplementary material for: Fairness in the multi-proposer-multi-responder ultimatum game
Source: PLoS One. 2025 Mar 4;20(3):e0319178. doi: 10.1371/journal.pone.0319178 (PMC11878916; doi:10.1371/journal.pone.0319178)
Supplement: S1 Appendix — We expand on the mathematical derivations and technical steps that were briefly outlined in the core sections of the paper. We provide detailed proofs and theorems that support the results presented in the main text. (PDF) [file pone.0319178.s001.pdf]

Supplementary Information: Fairness in the Multi-Proposer-Multi-Responder Ultimatum Game

Hana Krakovská,[1,2,*] Rudolf Hanel,[1,2] and Mark Broom[3]

[1]*Institute of the Science of Complex Systems, Center for Medical Data Science, Medical University of Vienna, Spitalgasse 23, Vienna, 1090, Austria*
[2]*Complexity Science Hub, Metternichgasse 8, Vienna, 1030 Austria*
[3]*Department of Mathematics, City, University of London, Northampton Square, London, EC1V 0HB, United Kingdom*

In this Supplementary Information, we will prove and show several key theorems and observations that are utilized in the main part of the manuscript. In the following section we start by proving that two vs. two Multi-Proposer-Multi-Responder Ultimatum Game (MPMR UG) has a unique evolutionarily stable strategy. We also demonstrate that under replicator dynamics with a population composed of players playing Nash equilibria, the population in a stable state plays the derived evolutionarily stable strategy, albeit on a population-wide level. In "General Case", we extend the results from two vs. two MPMR UG to arbitrary numbers of multiple responders and multiple proposers and show that also, in this case, there is a unique evolutionarily stable strategy for any subgame (apart from pure zero offers). Furthermore, we prove that if responders play this ESS Nash equilibrium in each subgame then in the subgame-perfect Nash equilibrium proposers have to propose the same offers.

TWO PROPOSERS AND TWO RESPONDERS

In this section, we will prove results concerning two vs. two MPMR UG (refer to the section with the same title in the main manuscript). We will prove that the symmetric strategy

$$p_A = \frac{2s_1 - s_2}{s_1 + s_2}\ , \tag{1}$$

where s_1, s_2 are offers which are similar enough ($s_2 < 2s_1$), is an evolutionarily stable strategy. Then in Replicator Dynamics we demonstrate that under replicator dynamics with three different Nash equilibrium strategy types, the solutions on the stable line of equilibria form the mixed strategy p_A, albeit on a population level.

Evolutionarily Stable Strategy

First, we start with the definition of an evolutionarily stable strategy.

Definition 1. *Let us denote by $\Pi(X,Y)$ the payoff of a player playing strategy X against strategy Y. Strategy X is called evolutionarily stable if for any $X \neq Y$:*

1. $\Pi(X,X) > \Pi(Y,X)$,

or

2. $\Pi(X,X) = \Pi(Y,X)$ *and* $\Pi(X,Y) > \Pi(Y,Y)$.

In the following theorem we prove that strategy p_A (see Eq. (1)) is a unique evolutionarily stable strategy.

Theorem 1. *Consider MPMR UG with two proposers and two responders. Consider proposers' offers, denoted as s_1, s_2, where $0 < s_1 \leq s_2 \leq 2s_1$ and $s_2 \leq 1$. Then, the mixed strategy $(p_A, 1-p_A)$, where $p_A = \dfrac{2s_1 - s_2}{s_1 + s_2}$ is the probability of choosing the first proposer, is a unique evolutionarily stable strategy for each subgame defined by offers s_1, s_2.*

---

* Corresponding author:hana.krakovska@savba.sk

Proof. We denote the strategy $(p_A, 1-p_A)$ as A and prove it is evolutionarily stable according to the second condition of Definition 1, we also refer to the notation introduced there. Let us rewrite the offers as $s_1 = s$ and $s_2 = s+\delta$, where $0 \le \delta \le s$. It is easy to see (see the manuscript for details) that:

$$\Pi(A,A) = \frac{3s(s+\delta)}{2(2s+\delta)} \quad \text{and} \quad \Pi(Y,A) = \frac{3s(s+\delta)}{2(2s+\delta)} \quad \text{for any strategy } Y \ ,$$

which proves the first property of the second condition. Next, we prove that

$$\Pi(A,Y) > \Pi(Y,Y) \text{ for any strategy } Y \neq A \ .$$

Let us represent strategy Y as having the probability $p \in [0,1]$ of going to the first proposer and probability $1-p$ of going to the second proposer. Then

$$\Pi(A,Y) - \Pi(Y,Y) = \frac{(\delta - s + \delta p + 2ps)^2}{2(2s+\delta)} \ , \tag{2}$$

where the equivalence is reached when

$$\delta - s + \delta p + 2ps = 0 \quad \Longleftrightarrow \quad p = \frac{s-\delta}{2s+\delta} \ .$$

Since strategy $p = \frac{s-\delta}{2s+\delta}$ is in fact strategy A, we showed that $\Pi(A,Y) > \Pi(Y,Y)$ for any strategy $Y \neq A$. Additionally, strategy A is uniquely ESS, since for any strategy $X \neq A$, we can not have $\Pi(X,X) \ge \Pi(Y,X)$ because $\Pi(X,X) < \Pi(A,X)$. □

Replicator Dynamics

In this subsection, we analyse the replicator dynamics involving three types of responders, each corresponding to a different Nash equilibrium strategy. We demonstrate that under the replicator dynamics the abundance ratios in the stable states correspond to the strategy p_A (see Eq. (1)), albeit on a population level.

Let us consider three types of players: γ–players that always play evolutionarily stable strategy p_A, α–players who always choose the higher offer, β–players who always choose the smaller offer. If the offers are the same, α and β players choose randomly one of them. We denote the fractions of γ–players, α–players, and β–players in the population as x_1, x_2, and x_3, respectively, where $x_1 + x_2 + x_3 = 1$. We use the same notation as in Theorem 1, where the offers are denoted as s_1 and s_2, with $s_1 = s$ and $s_2 = s+\delta$, where $0 \le \delta \le s$. Using the payoff matrix presented in Table 1 in the main manuscript, denoted here as P, we can construct the equations of the replicator dynamics:

$$\dot{x}_i = x_i \left[(Px)_i - x^T P x \right] \quad \text{for } i \in \{1,2,3\} \ ,$$

which gives us

$$\begin{aligned} \dot{x}_1 &= x_1 \frac{(x_1(2\delta+s) + x_2(\delta+2s) - 2\delta - s)^2}{2\delta+4s}, \\ \dot{x}_2 &= x_2 \frac{\begin{array}{r}\delta^2(4x_1^2 + 4x_1x_2 - 6x_1 + x_2^2 - 3x_2 + 2) + \delta s(4x_1^2 + 10x_1x_2 - 9x_1 + \\ 4x_2^2 - 9x_2 + 5) + s^2(x_1^2 + 4x_1x_2 - 3x_1 + 4x_2^2 - 6x_2 + 2)\end{array}}{2\delta+4s} \ , \end{aligned} \tag{3}$$

where $\dot{x}_3 = -\dot{x}_1 - \dot{x}_2$.

Let us analyse the stability of the system. It is easy to show that there are two equilibria $\{0,0,1\}$, $\{0,1,0\}$ and a line of equilibria $l^* = \{1 - x_2\frac{\delta+2s}{2\delta+s}, x_2, x_2\frac{s-\delta}{2\delta+s}\}$. The linearisation of the system at equilibria $\{0,0,1\}$ and $\{0,1,0\}$ reveals that both equilibria are unstable.

Linearisation at the line reveals that the equilibria have one zero and one negative eigenvalue. As seen in the numerically analysed dynamics (see Figure 1) the equilibrium line l^* is globally attracting on the whole simplex apart from the two unstable equilibria.

In the stable equilibrium line case l^* the expected payoffs of responders are:

$$\Pi(\alpha) = \Pi(\beta) = \Pi(\gamma) = \frac{3s(\delta+s)}{2(\delta+2s)} \ , \tag{4}$$

and the expected probability of going to the lower offer proposer in the mixed case on the line is $\frac{s-\delta}{\delta+2s}$ which is the same probability as in strategy p_A.

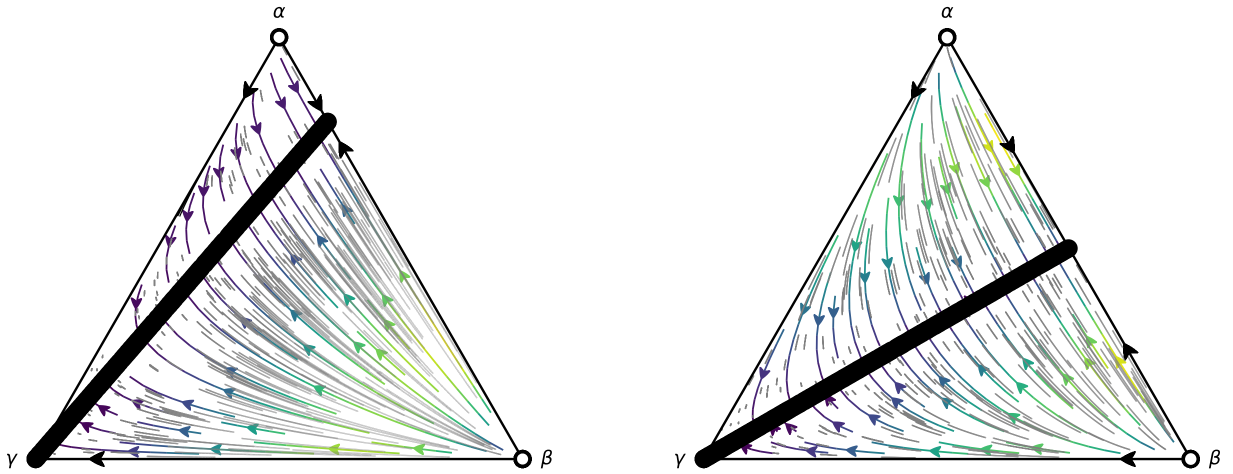

FIG. 1. Phase-space of the replicator dynamics (see Eq. (3)) for $s = 0.2, \delta = 0.1$ (left) and $s = 0.2, \delta = 0$ (right). Light yellow stands for high gradient values, and deep purple for low. Figures were generated with software made by Fernández [1].

GENERAL CASE

In this section we provide proofs of propositions and theorems needed in the main manuscript (section named "K Proposers and L Responders"). In the first subsection we show that there exists a unique evolutionarily stable strategy for any offer combination, apart from all offers being zero. In the second subsection we prove that if responders play this evolutionarily stable strategy, then the proposers' equilibrium strategy must be symmetric.

We start by repeating the notation from the main manuscript. We consider $K \geq 2$ proposers and $L \geq 2$ responders. The strategy of each proposer that describes how much they offer to the responders is denoted as $s_i \in [0, 1]$, $i \in \{1, 2, \dots, K\}$, and for every responder we allow a mixed strategy denoted:

$$p_{l,i} \geq 0,\ l \in \{1, 2, \dots, L\},\ i \in \{1, 2, \dots, K\},\ \text{s.t. for all } l : \sum_{i=1}^{K} p_{l,i} = 1\ ,$$

which determines the probability of the responder l choosing the proposer i. Note that the responder may reject all offers with some probability. In this case, the sum of the visitation probabilities does not necessarily equal one. However, to identify Nash equilibria of responders in each subgame, we can exclude these strategies if at least one offer is greater than zero. Let us denote $\Pi_{R,l}$ the expected payoff of responder l. We call the following system of equations *System R*:

For $l \in \{1, 2, \dots, L\}$ and $i \in \{1, 2, \dots, K-1\}$:

$$\begin{aligned} &\frac{\partial \Pi_{R,l}}{\partial p_{li}} = s_i W_{l,i} - s_K W_{l,K}\ , \\ &\text{where for all } m \in \{1, 2, \dots, K\} : \quad W_{l,m} = \sum_{j=1}^{L} \frac{1}{j} \left[\sum_{\alpha : \|\alpha\|_1 = j, \alpha_l = 1} \prod_{k=1, k \neq l}^{L} p_{km}^{\alpha_k} (1 - p_{km})^{1-\alpha_k} \right] . \end{aligned} \tag{5}$$

If all other responders apart from responder L use the same strategy $p_i, i \in \{1, 2, \dots, K\}$, where $\sum_{i=1}^{K} p_i = 1$ and $p_i \geq 0$, then the derivatives of the payoff of responder L can be simplified to *System S*:
For $i \in \{1, 2, \dots, K-1\}$:

$$\begin{aligned} &\frac{\partial \Pi_{R,L}}{\partial p_{Li}} = \frac{1}{L}(s_i f(p_i) - s_K f(p_K))\ , \\ &\text{where } f : [0,1] \to \mathbb{R}, \quad f(x) = \frac{1 - (1-x)^L}{x} \quad \text{and} \quad f(0) = L\ . \end{aligned} \tag{6}$$

Evolutionarily Stable Strategy

In this section we prove Theorem 2 which states that for any set of proposer offers (apart from all proposers offering zero) there exists a unique evolutionarily stable strategy of responders. We refer to the definition of evolutionarily stable strategy in multiplayer games by Broom et al. [2].

Definition 2. *Consider a multiplayer game with m players. Strategy A is evolutionarily stable against strategy Y if and only if there is $j \in \{0, 1, \ldots, m-1\}$ such that*

$$\Pi(A; A^{m-1-j}, Y^j) > \Pi(Y; A^{m-1-j}, Y^j),$$
$$\Pi(A; A^{m-1-i}, Y^i) = \Pi(Y; A^{m-1-i}, Y^i) \text{ for all } i < j,$$

where $\Pi(A; X_1, X_2, \ldots, X_{m-1})$ denotes payoff of player playing strategy A against $m-1$ players playing strategies $X_i, i \in \{1, 2, \ldots, m-1\}$ and X^i denotes that i players use the same strategy X. Strategy A is called an evolutionarily stable strategy at level J if, for every $Y \neq A$ the conditions above are satisfied for some $j \leq J$ and there is at least one $Y \neq A$ for which the conditions are met for $j = J$ precisely.

First we prove two simple lemmas which will be needed for the proof of Theorem 2. In the first lemma we show some basic properties of a central function in the derivatives of responders' payoffs (Eq. (6)).

Lemma 1. *Consider $L \geq 2$ and function*

$$f : \mathbb{R} \to \mathbb{R}, \quad f(x) = \frac{1-(1-x)^L}{x} \quad \text{and} \quad f(0) = L \ .$$

Then f is strictly decreasing, continuous and positive on $[0, 1]$.

Proof. It is easy to show with l'Hôpital rule that $\lim_{x\to 0} f(x) = L$ which gives us that the function $f(x)$ is continuous. Next, we want to show that the function's derivative is always negative on $(0, 1]$:

$$f'(x) = \frac{Lx(1-x)^{L-1} + (1-x)^L - 1}{x^2} \ .$$

As we prove in Lemma 3 function $-f'$ is a positive function, meaning the derivative f' is a negative function, thus f is decreasing, it is also positive on $[0, 1]$, since $f(1)$ is 1. □

In the following lemma, we prove a positive definiteness of a specific matrix, needed for a proof of convexity in Theorem 2.

Lemma 2. *Consider $K \times K$ symmetric matrix H with off-diagonal elements $H_{ij} = C, C > 0$ for $i \neq j$ and diagonal elements $H_{ii} = C + \varepsilon_i$, where $\varepsilon_i > 0$ for all $i \in \{1, 2, \ldots, K\}$. Then H is positive-definite.*

Proof. Matrix H is said to be positive-definite if

$$x^T H x > 0 \text{ for any vector } 0 \neq x \in \mathbb{R}^K \ . \tag{7}$$

We have that

$$x^T H x = \sum_{i=1}^{K} (C + \varepsilon_i) x_i^2 + \sum_{i,j=1, i\neq j}^{K} C x_i x_j \ . \tag{8}$$

It is clear that if $\sum_{i,j=1,i\neq j}^{K} C x_i x_j \geq 0$ the condition in (7) is satisfied. Now, let us assume $\sum_{i,j=1,i\neq j}^{K} C x_i x_j < 0$ and set $\varepsilon_{min} = \min_{i\in\{1,2,\ldots,K\}} \varepsilon_i$. Then we can rewrite (8) as

$$x^T H x = (C + \varepsilon_{min}) \left[\left(\sum_{i=1}^{K} x_i \right)^2 - \sum_{i,j=1,i\neq j}^{K} x_i x_j \right] + \sum_{i=1}^{K} (\varepsilon_i - \varepsilon_{min}) x_i^2 + \sum_{i,j=1,i\neq j}^{K} C x_i x_j \ ,$$

which is positive since it equals

$$(C + \varepsilon_{min}) \left(\sum_{i=1}^{K} x_i \right)^2 + \sum_{i=1}^{K} (\varepsilon_i - \varepsilon_{min}) x_i^2 - \varepsilon_{min} \sum_{i,j=1,i\neq j}^{K} x_i x_j \ ,$$

and we have $\sum_{i,j=1,i\neq j}^{K} C x_i x_j < 0$. □

Finally, we prove the main theorem of this subsection which states that for any offer combination (apart from all offers being zero) there exists a unique evolutionarily stable strategy of responders.

Theorem 2. *Consider MPMR UG with $K \geq 2$ proposers and $L \geq 2$ responders. Consider a set of offers $s_K \geq s_{K-1} \geq \cdots \geq s_1$, where $s_K \in (0,1]$ and $s_1 \geq 0$. Then for each given set of offers, there exists a unique evolutionarily stable strategy at level 1 of responders, implicitly defined for all $i \in \{1,2,\ldots,K-1\}, p_i \geq 0$ such that $\sum_{i=1}^{K} p_i = 1$ and $p_K > 0$ as:*

$$\begin{aligned} &p_i = 0 \qquad \text{and} \;\; s_i f(p_i) - s_K f(p_K) \leq 0 \;, \\ &\text{or} \\ &p_i > 0 \qquad \text{and} \;\; s_i f(p_i) - s_K f(p_K) = 0 \;\Rightarrow\; p_i = f^{-1}\left(\frac{s_K}{s_i} f(p_K)\right) , \end{aligned} \tag{9}$$

where $f : [0,1] \to \mathbb{R}, f(x) = \frac{1-(1-x)^L}{x}$ and $f(0) = L$.

Proof. Let us denote the solution p_i of Eq. (9) as strategy A. As was shown in the analysis in "K Proposers and L Responders", such solution always exists and is unique for any set of offers (apart from pure zero offers). First, let us assume we have a set of offers that results in strategy A following the equivalence for all $i : s_i f(p_i) - s_K f(p_K) = 0$. Now refer to the previous Definition 2, for $j = 0$ we have to show that

$$\Pi(A; A^{L-1}) = \Pi(Y; A^{L-1}) \; .$$

This is evident from the fact that the derivatives of the first responder's payoff with respect to their strategies, $\frac{\partial \Pi_{R,1}}{\partial p_{1i}}$, are independent of their strategy p_{1i} and equal to zero when all other players use strategy A (see *System S* in (6)).

Next, we look at $j = 1$. We need to show that

$$\Pi(A; A^{L-2}, Y) > \Pi(Y; A^{L-2}, Y) \; .$$

Let us denote the strategy Y as $q_i, i \in \{1,2,\ldots,K\}$. We may rewrite the payoffs of the players using strategy A and Y (denoted Π_A and Π_Y respectively) as:

$$\begin{aligned} \Pi_A &= \sum_{i=1}^{K} s_i p_i \left[q_i A_{1,L}(i) + (1-q_i) B_{1,L}(i) \right] \;, \\ \Pi_Y &= \sum_{i=1}^{K} s_i q_i \left[q_i A_{1,L}(i) + (1-q_i) B_{1,L}(i) \right] \;, \end{aligned}$$

where

$$\begin{aligned} A_{1,L}(i) &= \sum_{j=2}^{L} \frac{1}{j} \sum_{\alpha:\|\alpha\|_1 = j, \alpha_1 = 1, \alpha_L = 1} \prod_{k \neq 1,L} p_i^{\alpha_k} (1-p_i)^{1-\alpha_k} \;, \\ B_{1,L}(i) &= \sum_{j=1}^{L-1} \frac{1}{j} \sum_{\alpha:\|\alpha\|_1 = j, \alpha_1 = 1, \alpha_L = 0} \prod_{k \neq 1,L} p_i^{\alpha_k} (1-p_i)^{1-\alpha_k} \;, \end{aligned}$$

and if number of responders is two then

$$A_{1L}(i) = \frac{1}{2} \quad \text{and} \quad B_{1L}(i) = 1 \; .$$

From this we have

$$\Pi_A - \Pi_Y = \sum_{i=1}^{K} s_i (p_i - q_i) \left[q_i A_{1,L}(i) + (1-q_i) B_{1,L}(i) \right] .$$

Then $\Pi_A - \Pi_Y = 0$ for $q = p$. From the way the strategy A is defined we have that

$$s_i \left[p_i A_{1,L}(i) + (1-p_i) B_{1,L}(i) \right] - s_K \left[p_K A_{1,L}(K) + (1-p_K) B_{1,L}(K) \right] = 0 \; . \tag{10}$$

In the following we will show that the point $q = p$ is a global minimum of $\Pi_A - \Pi_Y$. We start with the derivatives, remember $q_K = 1 - \sum_{i=1}^{K-1} q_i$:

$$\frac{\partial(\Pi_A - \Pi_Y)}{\partial q_i} = s_i \left[-B_{1,L}(i) + (p_i - 2q_i)(A_{1,L}(i) - B_{1,L}(i))\right] - s_K \left[-B_{1,L}(K) + (p_K - 2q_K)(A_{1,L}(K) - B_{1,L}(K))\right] \ .$$

For the derivative of the payoff difference where $p = q$ we get:

$$\left.\frac{\partial(\Pi_A - \Pi_Y)}{\partial q_i}\right|_p = s_i \left[-B_{1,L}(i) - p_i(A_{1,L}(i) - B_{1,L}(i))\right] - s_K \left[-B_{1,L}(K) - p_K(A_{1,L}(K) - B_{1,L}(K))\right] \ ,$$

which is clearly equal to zero since equation (10) is equal to zero. Next, we look at the second derivatives

$$\frac{\partial^2(\Pi_A - \Pi_Y)}{\partial q_i q_i} = -2s_i(A_{1,L}(i) - B_{1,L}(i)) - 2s_K(A_{1,L}(K) - B_{1,L}(K)) \ , \tag{11}$$

and for $i \neq j$

$$\frac{\partial^2(\Pi_A - \Pi_Y)}{\partial q_i q_j} = -2s_K(A_{1,L}(K) - B_{1,L}(K)) \ . \tag{12}$$

We know that all the elements of the Hessian matrix (11) and (12) are positive, since $A_{1,L}(i) - B_{1,L}(i) < 0$ for all $i \in \{1, 2, \dots, K\}$ and also all s_i must be positive (since we consider the equivalence case for all i). Thus, the Hessian is symmetric with equal positive off-diagonal entries and diagonal entries $H_{ii} > H_{jk}$ for all $i, j, k \in \{1, 2, \dots, K-1\}$. It is easy to prove that such matrix is positive definite (see Lemma 2). The second derivative is also independent of q and thus we see that the function $\Pi_A - \Pi_Y$ is strictly convex in q. This means that the minimum at $q = p$ is also a unique global minimum. From this it follows that

$$\Pi_A = \Pi(A; A^{L-2}, Y) > \Pi(Y; A^{L-2}, Y) = \Pi_Y \ .$$

Thus, we have shown that strategy A is a unique ESS in the equivalence to zero case.

Next, let us assume that not all derivatives are equal to zero, but there are some $i \in I$, where $I \subseteq \{1, 2, \dots, K-1\}$ for which the derivative is negative and therefore $p_i = 0$. Now, there are two groups of strategies Y we have to look at. In the first group there are strategies $\{q_i\}_{i=1}^K$ such that $q_i = 0$, for all $i \in I$. In this case we can discard all the offers s_i for $i \in I$ and consequently prove the evolutionary stability in the same way as in the previous part of the proof.

In the second group, there are strategies for which $q_i \neq 0$ for some $i \in I$. Then, due to the form of the derivatives we must have already for $j = 0$

$$\Pi(A; A^{L-1}) > \Pi(Y; A^{L-1}) \ .$$

□

Proposers' Subgame-Perfect Nash Strategy

In the following subsection, we prove an important result necessary for the analysis of the general case (see "K Proposers and L Responders" in the main manuscript), which states that if responders follow their evolutionarily stable strategy, the proposers' Nash equilibrium must be symmetric. Before proving the theorem, we introduce two lemmas that demonstrate properties of two functions essential for the proof of Theorem 3 and Proposition 1, where we prove that the second derivative of the payoff at the proposer's equilibrium is negative.

Lemma 3. *Consider $L \geq 2$ and function*

$$f : \mathbb{R} \to \mathbb{R} \ , \ f_L(x) = \frac{1 - (1-x)^L - Lx(1-x)^{L-1}}{x^2} \ \text{and} \ f_L(0) = \frac{L(L-1)}{2} \ .$$

Then f is continuous, positive and constant function for $L = 2$, and decreasing function for $L > 2$ on interval $[0, 1]$.

Proof. Continuity is trivial to show. We will prove the other properties by mathematical induction. We start with $L = 2$

$$f_2(x) = \frac{1-(1-x)^2-2x(1-x)}{x^2} = 1 \ ,$$

f_2 is a constant function which is non-increasing. Now, we assume that for f_N is non-increasing and look at $L = N+1$, after some manipulations we arrive to:

$$f_{N+1}(x) \quad = f_N(x) + N(1-x)^{N-1} \ ,$$

since f_{N+1} is a sum of a non-increasing functions and a decreasing function, for $L > 2$, f_L is decreasing. Since it is decreasing, we know that minimum on the interval $[0, 1]$ is at 1 where $f_L(1) = 1$. Thus f_L is a positive function. □

Lemma 4. *Consider $L \geq 2$ and functions*

$$f : \mathbb{R} \to \mathbb{R} \ , \ f_L(x) = \frac{Lx^2(1-x)^{L-1}}{1-(1-x)^L - Lx(1-x)^{L-1}} \ , \ f_L(0) = \frac{2}{L-1} \ ,$$

Then f_L is decreasing function on $(0, 1]$ for $L > 1$. It is also positive on $[0, 1)$ and $f(1) = 0$.

Proof. We will prove that the derivative of the function $f'_L(x)$ is negative on $(0, 1]$ for any L.

$$f'_L(x) = \frac{Lx(1-x)^{L-2}((-Lx+x-2)(1-x)^L - Lx - x + 2)}{(1-(1-x)^L - Lx(1-x)^{L-1})^2} \ .$$

We see it is sufficient to show that

$$(-Lx + x - 2)(1-x)^L - Lx - x + 2 < 0 \ .$$

We prove it by mathematical induction. For $L = 2$ we have

$$f'_2(x) = -x^3 \ ,$$

which is negative on $(0, 1]$. Now, we assume it holds for $L = N$:

$$f'_N(x) = (-Nx + x - 2)(1-x)^N - Nx - x + 2 < 0 \ ,$$

notice we have that

$$(1-x)f'_N(x) = (-Nx + x - 2)(1-x)^{N+1} - Nx - x + 2 + Nx^2 + x^2 - 2x < 0 \ .$$

When we look at $L = N + 1$ we can rewrite f'_{N+1} as:

$$f'_{N+1}(x) = (-(N+1)x + x - 2)(1-x)^{N+1} - (N+1)x - x + 2 = (1-x)f'_N(x) + x\left(1 - (N+1)x - (1-x)^{N+1}\right) \ .$$

Function $r(x) = 1-(N+1)x-(1-x)^{N+1}$ is non-positive. At zero we have $r(0) = 0$ and then the derivative is clearly negative for $x \in (0, 1]$. Thus, $f'_{N+1}(x)$ is a sum of two negative functions and therefore it is also negative. This means f_L is a decreasing function on $(0, 1]$. Additionally, $f_L(1) = 0$ and therefore f_L is positive on $[0, 1)$. □

After introducing the lemmas, we can proceed with the main theorem, which states that under the responders' evolutionarily stable strategy, the subgame-perfect Nash equilibrium must be symmetric.

Theorem 3. *Consider a MPMR UG with $K \geq 2$ proposers and $L \geq 2$ responders. If all responders follow the evolutionarily stable strategy described above and all offers are greater than zero, then the subgame-perfect Nash equilibrium of proposer strategies must be symmetric i.e. for all $i, j \in \{1, 2, \ldots, K\}$: $s_i = s_j$.*

Proof. It is clear that if an offer leads to proposer's overall selection probability zero this can not constitute a Nash equilibrium, since by raising the offer to the highest offer (or in the case the highest offer is one then a sufficiently smaller offer than one) the proposer can reach a non-zero payoff. Thus, for Nash equilibria candidates we will only consider those sets of offers $\{s_1, s_2, \ldots, s_K\}$ for which the evolutionarily stable strategy yields $p_i > 0$ for all proposers $i \in \{1, 2, \ldots, K\}$. If this is the case then also a feasible solution to the homogeneous system in (6) exists.

Let us remind that selection probabilities of proposers, $p_i(s_1, s_2, \dots, s_K)$ for $i \in \{1, 2, \dots, K\}$, are functions of the offer set. The payoff of the proposer i is

$$\Pi_{P,i} = (1 - s_i)\left(1 - (1 - p_i)^L\right), \tag{13}$$

and due to $\frac{\partial \Pi_R}{\partial p_i} = 0$ we have that

$$\frac{s_i}{p_i}\left(1 - (1 - p_i)^L\right) = \frac{s_K}{p_K}\left(1 - (1 - p_K)^L\right) =: C(s_1, s_2, \dots s_K) \ . \tag{14}$$

Thus, we can then rewrite the payoff in (13) as

$$\Pi_{P,i} = 1 - (1 - p_i)^L - C p_i \ .$$

In Nash equilibrium we need the derivative of the proposer's payoff with respect to the strategy s_i to be zero:

$$\frac{\partial \Pi_{P,i}}{\partial s_i} = L(1 - p_i)^{L-1}\frac{\partial p_i}{\partial s_i} - C\frac{\partial p_i}{\partial s_i} - \frac{\partial C}{\partial s_i} p_i \ . \tag{15}$$

We denote $f(p_i) = \frac{1-(1-p_i)^L}{p_i}$ and have that $f(p_i) = \frac{C}{s_i}$, thanks to the inverse function theorem:

$$\begin{aligned} \frac{\partial p_i}{\partial s_i} &= \left.\frac{\partial f^{-1}}{\partial \frac{C}{s_i}}\right|_{\frac{C}{s_i}} \frac{\partial \frac{C}{s_i}}{\partial s_i} = \frac{g_i}{s_i}\frac{\partial C}{\partial s_i} - g_i \frac{C}{s_i^2}, \\ \frac{\partial p_j}{\partial s_i} &= \left.\frac{\partial f^{-1}}{\partial \frac{C}{s_j}}\right|_{\frac{C}{s_i}} \frac{\partial \frac{C}{s_j}}{\partial s_i} = \frac{g_j}{s_j}\frac{\partial C}{\partial s_i} \quad \text{for } i \neq j, \end{aligned} \tag{16}$$

where $g_i = \frac{1}{f'(p_i)} = \frac{p_i^2}{(1-p_i)^{L-1}(Lp_i + 1 - p_i) - 1}$. Since we know that

$$\sum_{j=1}^{K} \frac{\partial p_j}{\partial s_i} = 0 \quad \text{we have that} \quad \frac{\partial C}{\partial s_i} = \frac{1}{H}\frac{g_i C}{s_i^2} \ , \tag{17}$$

where $H = \sum_{j=1}^{K} \frac{g_j}{s_j}$. By submitting (16) and (17) into (15) we get

$$\frac{\partial \Pi_{P,i}}{\partial s_i} = \frac{\partial p_i}{\partial s_i}\left(L(1 - p_i)^{L-1} - C\right) - \frac{\partial C}{\partial s_i} p_i = \left(\frac{g_i^2 C}{s_i^3 H} - \frac{g_i C}{s_i^2}\right)\left(L(1 - p_i)^{L-1} - C\right) - \frac{p_i g_i C}{s_i^2 H} \ . \tag{18}$$

In the subgame-perfect Nash equilibrium (18) has to be equal to zero for all $i \in \{1, 2, \dots, K\}$. Thus, we can simplify set of equations in (18) to

$$d_i := \left(\frac{g_i}{s_i} - H\right)\left(L(1 - p_i)^{L-1} - C\right) - p_i = 0 \ , \ \text{for all } i \in \{1, 2, \dots, K\} \ . \tag{19}$$

Next, we will show that if $p_i < p_j$ for some i, j then $d_i > d_j$ which proves that other than the symmetric solution of a system of equations (19) does not exist. First we rewrite $\frac{g_i}{s_i}$ by using (14) as

$$\frac{g_i}{s_i} = \frac{g_i(1 - (1 - p_i)^L)}{C p_i} \ ,$$

which can be further rewritten as

$$\frac{g_i}{s_i} = \frac{1}{C}\left(-p_i - \frac{L p_i^2 (1 - p_i)^{L-1}}{1 - (1 - p_i)^L - L p_i (1 - p_i)^{L-1}}\right) \ ,$$

from which we can derive that

$$H = \sum_{i=1}^{K} \frac{g_i}{s_i} = \frac{1}{C}\left(-1 - \sum_{i=1}^{K} a_i\right),$$

where $a_i = \frac{Lp_i^2(1-p_i)^{L-1}}{1-(1-p_i)^L - Lp_i(1-p_i)^{L-1}}$. Then we can rewrite d_i (see Eq. (19)) as

$$\left(1 - p_i + \sum_{j=1, j\neq i}^{K} a_j\right)\left(L(1-p_i)^{L-1} - C\right) - Cp_i = 0 \ ,$$

and derive

$$C(p_i) = \frac{(1 - p_i + \sum_{j=1,j\neq i}^{K} a_j)L(1-p_i)^{L-1}}{1 + \sum_{j=1,j\neq i}^{K} a_j} \ .$$

Now, we proceed to prove the statement by contradiction. Let us assume without loss of generality that $0 < p_1 < p_2 < 1$. Since $C(p_1) = C(p_2)$, we have

$$(1 + A + a_1)(1 - p_1 + A + a_2)(1 - p_1)^{L-1} = (1 + A + a_2)(1 - p_2 + A + a_1)(1 - p_2)^{L-1},$$

where $A = \sum_{j=3}^{K} a_j$, which means it should also be true that

$$(1 + A + a_1)\left[(1 - p_1 + A)(1 - p_1)^{L-1} + a_2(1 - p_1)^{L-1}\right] = (1 + A + a_2)\left[(1 - p_2 + A)(1 - p_2)^{L-1} + a_1(1 - p_2)^{L-1}\right],$$

but as we show in the next steps, this is not possible. Since $p_1 < p_2$ and function a_i is decreasing and positive on $[0, 1)$ (see Lemma 4) we must have $a_1 > a_2$. That means that

$$(1 + A + a_1) > (1 + A + a_2) \quad \text{and} \quad (1 - p_1 + A)(1 - p_1)^{L-1} > (1 - p_2 + A)(1 - p_2)^{L-1} \ .$$

But as we will show also

$$a_2(1 - p_1)^{L-1} \geq a_1(1 - p_2)^{L-1} \ ,$$

must hold. After submitting for a_i, we have

$$\begin{aligned} \frac{Lp_2^2(1-p_2)^{L-1}}{1-(1-p_2)^L - Lp_2(1-p_2)^{L-1}}(1-p_1)^{L-1} &\geq \frac{Lp_1^2(1-p_1)^{L-1}}{1-(1-p_1)^L - Lp_1(1-p_1)^{L-1}}(1-p_2)^{L-1} \\ \frac{p_2^2}{1-(1-p_2)^L - Lp_2(1-p_2)^{L-1}} &\geq \frac{p_1^2}{1-(1-p_1)^L - Lp_1(1-p_1)^{L-1}} \ . \end{aligned} \tag{20}$$

We know the last line above is true thanks to Lemma 3 which states that reciprocal of $\frac{p_2^2}{1-(1-p_2)^L - Lp_2(1-p_2)^{L-1}}$ is a positive and non-increasing function on $[0, 1]$ and for $L \geq 2$, meaning the function in the last line of (20) is non-decreasing. Thus, we showed that for any $p_1 < p_2$ we can not have $C(p_1) = C(p_2)$. □

We conclude this section with a technical proposition showing that the second derivative of the proposers' payoff is negative in the equilibrium found in the main manuscript (see section named "K Proposers and L Responders"), thus proving, that it is indeed a maximum.

Proposition 1. *Consider MPMR UG with $L \geq 2$ responders and $K \geq 2$ proposers. The second derivative of the proposer's payoff is negative at the equilibrium s^* (see main manuscript "K Proposers and L Responders", subsection named "Proposers' Strategy").*

Proof. Let us remind us that in the equilibrium $p = \frac{1}{K}$, $s^* > 0$ and the derivative is

$$\frac{\partial \Pi_{P,i}}{\partial s_i} = \frac{g_i C}{s_i^2 H}\left[\left(\frac{g_i}{s_i} - H\right)\left(L(1-p_i)^{L-1} - C\right) - p_i\right],$$

where $H = \sum_{i=1}^{K} \frac{g_i}{s_i}$ and $g_i = \frac{p_i^2}{(1-p_i)^{L-1}(Lp_i+1-p_i)-1}$ is a function of p_i that is negative and non-increasing (for the proof see Lemma 3). We know that $\frac{g_i C}{s_i^2 H} > 0$ thus, $f_i = \left(\frac{g_i}{s_i} - H\right)\left(L(1-p_i)^{L-1} - C\right) - p_i$ must be equal to zero in equilibrium s^*. Then

$$\left.\frac{\partial \Pi_{P,i}}{\partial s_i^2}\right|_{s=s^*} = \left.\frac{\partial \frac{g_i C}{s_i^2 H}}{\partial s_i}\right|_{s=s^*} \cdot 0 + \frac{g_i C}{s_e^2 H}\left.\frac{\partial f_i}{\partial s_i}\right|_{s=s^*},$$

where

$$\frac{\partial f_i}{\partial s_i} = -\sum_{j\neq i} \frac{g_j'}{s_j}\frac{\partial p_j}{\partial s_i}\left(L(1-p_i)^{L-1} - C\right) - \sum_{j\neq i}\frac{g_j}{s_j}\left(-L(L-1)(1-p_i)^{L-2}\frac{\partial p_i}{\partial s_i} - \frac{\partial C}{\partial s_i}\right) - \frac{\partial p_i}{\partial s_i}\ ,$$

where $g_i' = \frac{\partial g_i}{\partial p_i}$. Now it is easy to see that $\left.\frac{\partial f_i}{\partial s_i}\right|_{s=s^*} < 0$. The first term must be non-positive, since g_j' is non-positive, $\left.\frac{\partial p_j}{\partial s_i}\right|_{s=s^*} < 0$ for $i \neq j$ and $(L(1-p_i)^{L-1} - C) > 0$ in the equilibrium. The second and third term must be negative, since g_j is negative and both $\frac{\partial p_i}{\partial s_i}$ and $\frac{\partial C}{\partial s_i}$ are positive in the equilibrium. Lastly, also $\frac{g_i C}{s_i^2 H} > 0$, thus $\left.\frac{\partial \Pi_{P,i}}{\partial s_i^2}\right|_{s=s^*} < 0$. □

[1] E. Fernández Domingos, Egttools: Toolbox for evolutionary game theory, `https://github.com/Socrats/EGTTools` (2020).
[2] M. Broom and J. Rychtář, *Game-theoretical models in biology* (Chapman and Hall/CRC, 2022).
